# Prioritization of candidate disease genes by topological similarity between disease and protein diffusion profiles

**DOI:** 10.1186/1471-2105-14-S5-S5

**Published:** 2013-04-10

**Authors:** Jie Zhu, Yufang Qin, Taigang Liu, Jun Wang, Xiaoqi Zheng

**Affiliations:** 1 Department of Mathematics, Shanghai Normal University, Shanghai 200034, China; 2 College of Information Technology, Shanghai Ocean University, Shanghai 201306, China; 3 Scientific Computing Key Laboratory of Shanghai Universities, Shanghai 200234, China

## Abstract

**Background:**

Identification of gene-phenotype relationships is a fundamental challenge in human health clinic. Based on the observation that genes causing the same or similar phenotypes tend to correlate with each other in the protein-protein interaction network, a lot of network-based approaches were proposed based on different underlying models. A recent comparative study showed that diffusion-based methods achieve the state-of-the-art predictive performance.

**Results:**

In this paper, a new diffusion-based method was proposed to prioritize candidate disease genes. *Diffusion profile *of a disease was defined as the stationary distribution of candidate genes given a random walk with restart where similarities between phenotypes are incorporated. Then, candidate disease genes are prioritized by comparing their diffusion profiles with that of the disease. Finally, the effectiveness of our method was demonstrated through the leave-one-out cross-validation against control genes from artificial linkage intervals and randomly chosen genes. Comparative study showed that our method achieves improved performance compared to some classical diffusion-based methods. To further illustrate our method, we used our algorithm to predict new causing genes of 16 multifactorial diseases including Prostate cancer and Alzheimer's disease, and the top predictions were in good consistent with literature reports.

**Conclusions:**

Our study indicates that integration of multiple information sources, especially the phenotype similarity profile data, and introduction of global similarity measure between disease and gene diffusion profiles are helpful for prioritizing candidate disease genes.

**Availability:**

Programs and data are available upon request.

## Background

Elucidating the relationship between human genetic diseases and their causal genes is an important emerging topic in current systematic biology. Understanding the inherited basis of these interactions could both improve medical care and better understand gene functions, interactions, and pathways. Typically, a disease is associated with a linkage interval of 0.5-10 cM on the chromosome if single nucleotide polymorphisms (SNPs) in this interval are correlated with an increased probability to have the disease [1-3]. Then, these linkage intervals define a set of (up to several hundreds) candidate disease-causing genes [4,5].

With the rapid accumulation of different kinds of genomic data, a lot of computational methods for prioritizing candidate casual genes of a given phenotype were proposed at the beginning of the 21^st ^century. These methods are largely based on the similarity of characteristics of disease genes, including sequence-based features [6-8], expression patterns [9-11], and functional annotation data [12,13]. Despite their good performances, these methods suffer from some inherent limitations, e.g., the incomplete and false-positive disease-causal genes data, ambiguous boundary between different diseases, and highly heterogeneous of diseases.

Recently, network-based analysis showed that gene products related to the same disease are prone to physically interact with each other [14-17]. Based on this observation, a number of computational approaches have been proposed to predict associations between genes and diseases. These methods mainly begin with an artificial disease interval and test their ability to identity a real causing gene among a fixed number of nearby control genes. According to their underlying methodology, these methods can be loosely grouped into three categories [18]. The first category is the linkage methods, which assumed that the direct interaction partners of a disease protein are likely to associate with the same disease phenotype. It was found that gene products interacted with a known disease protein were shown to be tenfold enriched in true disease-causing genes [19], so many researchers searched the PPI network for direct or indirect interacting partners of known disease genes to find new possible causing genes [10,20,21]. The second category is module-based methods, which are based the observation that gene products belonging to the same topological, functional or disease module have a high likelihood of being involved in the same disease. These methods inspect disease modules by graph partition algorithms and treat their members as potential disease genes [22]. The last category consists of diffusion-based methods [23-26]. In these algorithms, 'random walkers' are released from the protein products of known disease genes, and then diffuse along the PPI network, with a certain probability to return the original nodes. Compared to linkage-based and module-based methods, diffusion-based methods used information encoded in the full network topology as well as the placement of all known disease genes. So a recent comparative research found that diffusion-based methods achieved the best predictive performance on the same data set [27].

In the present paper, we propose a new diffusion-based method to prioritize candidate disease genes. The *diffusion profile *of a disease was defined as the stationary distribution of all candidate genes in the PPI network under a random walk with restart where similarities between phenotypes are incorporated. Similarly, the diffusion profile of a gene was obtained by smoothing the probability distribution over the whole network when starting a walk from this gene. Then, candidate disease genes are prioritized by comparing their diffusion profiles with that of the disease, measured by the linear correlation coefficient and cosine of the angle between profile vectors. Finally, the effectiveness of our method was demonstrated through the leave-one-out cross-validation against control genes from artificial linkage intervals and randomly chosen genes. Comparisons of our method with two classical diffusion-based methods showed that our method achieves improved performance. To further illustrate our method, we also used our algorithm to predict new causing genes of 16 multifactorial diseases including prostate cancer and Alzheimer's disease, and our top predictions are in good consistent with literature reports.

## Methods

### Protein-protein interaction data and known disease-gene associations

The protein-protein interaction network (PPI) is modelled as an undirected graph with nodes representing the genes and edges representing the physical or binding interactions between proteins encoded by the genes. In the present paper, PPI network is obtained from release 9 of the Human Protein Reference Database (HPRD) [28]. After removing duplications and self-linked interactions, we obtain 37 064 manually curated interactions between 9515 human genes.

Disease-gene association data are downloaded from the Online Mendelian Inheritance in Man (OMIM) knowledgebase [29]. The dataset contains 2704 diseases and 5316 disease-gene associations after removing the duplications, with an average of 1.97 gene associations for each disease. To facilitate the cross-validation, diseases currently associated with only one causal gene were discarded. Meanwhile, associations not correlated with the 9515 human genes in the PPI network were also excluded. After these steps, a total number of 1238 validate disease-gene associations are left for further consideration.

**Figure 1:**
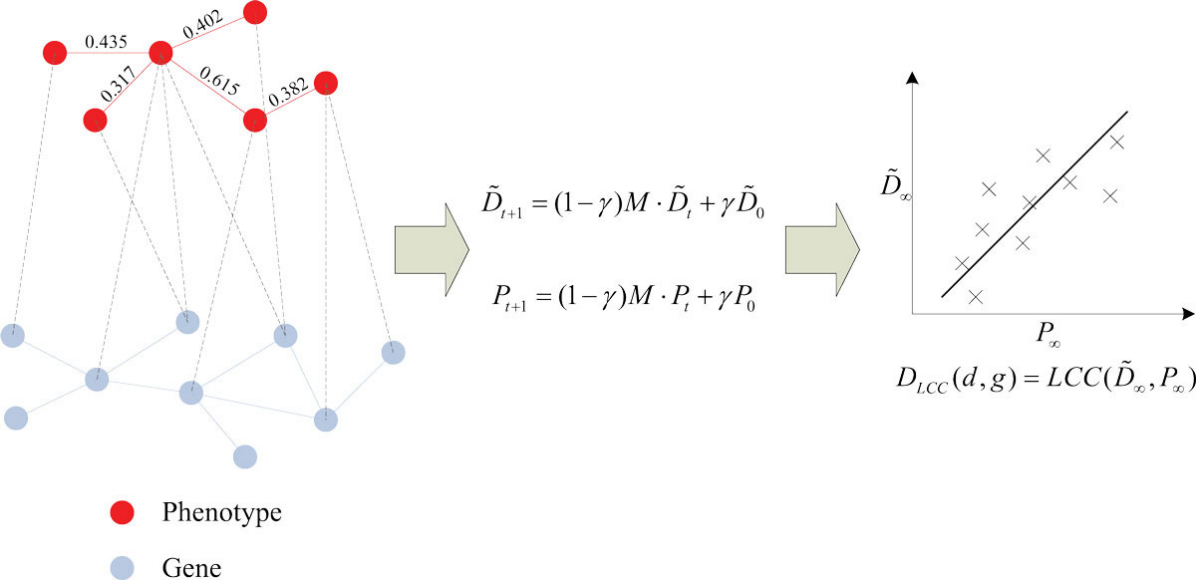
**Workflow of the DP_LCC method**. i) The phenome-interactome network is constructed by integrating the PPI network, disease-gene associations and currently known phenotype similarity data. ii) Diffusion profiles of diseases and genes are calculated by a random walk over the PPI network with restart where similarities between phenotypes are incorporated. iii) Candidate genes are then prioritized according to their similarities with the disease diffusion profile. The numbers on the edges of phenome network indicate their pairwise similarities.

Phenotype similarities between 5080 disease phenotypes obtained from the literature [30] are incorporated for prioritization of candidate disease genes. These similarities are calculated by text mining of OMIM phenotype records using Medical Subject Headings (MeSH) terms [31]. According to their analysis, similarity values below the threshold 0.3 are not informative, while for similarity values beyond 0.6 the associated genes show significant functional similarity. So in our analysis, similarity values below 0.3 are not considered for the computation of the diffusion profile of a disease (Figure 1).

### Random walk with restart and diffusion profile of a protein

Given a PPI network G=<V,E> where  V is the set of proteins,  E is the set of interactions. The random walk on PPI network is defined as an iterative walker's transition from its current node to any neighbouring node with equal probability starting at a given source node(s). In a statistical point of view, a random walk is a finite Markov chain that is time-reversible.

For example, if a random walk begins with a node g∈V, the initial distribution $P_{0}$ of the random walk was formulated as a vector of dimension ||V||, in which the element in position corresponding to *v *was 1 and 0 otherwise. The rule of the walk can be expressed by the equation

(1) $$P_{t+1}=M\cdot P_{t}\;t=0,1,2,\cdots \;$$

where *M *is the column-normalized adjacency matrix of the graph. After a certain steps, the probability will reach a *steady *(or *stationary*) *state*. This can be implemented by performing the iteration until the difference between $P_{t+1}$ and $P_{t}$ (measured by the $L_{1}$ norm) fall below $\text{1}{\text{0}}^{\text{ - 6}}$, i.e.,

(2) $$|P_{t+1}-P_{t}|<10^{-6}$$

If the walk has a certain probability  γ to return the start node at each step, that is,

(3) $$P_{t+1}=\left(1-\gamma \right)M\cdot P_{t}+\gamma P_{0}\;t=0,1,2,\cdots \;$$

which is the *random walk with restart*. The restart probability  γ enforces a restriction on how far we want the random walker to get away from the start node  V. If  γ is close to 1, the stationary probability vector reflects the local structure around the  V, and as  γ gets close to 0, a more global view is observed. Finally, we define the *diffusion profile of a protein g *as the stationary distribution $P_{\infty }$ of the random walk with the initial distribution$P_{0}$.

Random walk with restart (RWR) provides a good relevance between two nodes in a graph, and it has been successfully used in numerous settings, like automatic captioning of images, generalizations to the "connection subgraphs", personalized PageRank, and so on [32]. The main advantage of the random walk method is that its computational complexity is relatively low and applicable to handle large PPI networks. Moreover, the method can be used to compute the proximity of a node to a set of source nodes (not just a single source node). This property is especially beneficial when a core set of members of a phenotype is known and the network is queried for candidate members.

### Diffusion profile of a disease by incorporating OMIM Phenotype similarity and prioritizing function

Similarly, in order to compute the diffusion profile of a disease, we should first get the initial distribution of a disease. Here, the initial probability vector $D_{0}$ of a disease  d was constructed such that equal probability was assigned to each node representing the causing gene of the disease, with the sum of the probabilities equals 1. Then

(4) $$D_{t+1}=\left(1-\gamma \right)M\cdot D_{t}+\gamma D_{0}\;t=0,1,2,\cdots \;$$

In this way, proteins that interact with several disease proteins will gain a high probabilistic weight, as well as those that may not directly interact with any disease proteins but are in close network proximity to them.

It was found by researchers that similar phenotypes are caused by functionally related genes. Based on this observation, for a disease *d*, we used initial distributions of phenotypes which have similarity exceeds a threshold with *d *to optimize its initial distribution. We assume that the contribution of a given disease $d^{i}$ is proportional to the initial distribution of *d*. So the weighted initial distribution of the disease *d *is formulated as

(5) $${\tilde{D}}_{0}=D_{0}+\lambda \cdot \overset{5080}{\underset{i=1}{\sum }}\delta \left(d,d^{i}\right)\cdot Sim\left(d,d^{i}\right)\cdot D_{0}^{i}$$

where $\delta \left(d,d^{i}\right)=\left\{\begin{array}{cc} 1 & Sim\left(d,d^{i}\right)\geq 0.3\\ 0 & \text{otherwise} \end{array}\right)$, $D_{0}^{i}$ is the initial distribution of disease $d^{i}$.

Then

(6) $${\tilde{D}}_{t+1}=\left(1-\gamma \right)M\cdot {\tilde{D}}_{t}+\gamma {\tilde{D}}_{0}\;t=0,1,2,\cdots \;$$

We called the stationary distribution ${\tilde{D}}_{\infty }$ of the random walk *diffusion profile of the disease d*.

Finally, for a given disease and a set of genes in the PPI network, the prioritizing function can be defined by the Linear Correlation Coefficient (LCC, Figure 1) or Cosine angle of their corresponding diffusion profiles. Explicitly,

(7) $$D_{LCC}\left(d,g\right)=LCC\left({\tilde{D}}_{\infty },P_{\infty }\right)=\frac{\overset{n}{\underset{i=1}{\sum }}\left({\tilde{D}}_{\infty }\left(i\right)-\bar{{\tilde{D}}_{\infty }}\right)\left(P_{\infty }\left(i\right)-\bar{P_{\infty }}\right)}{\sqrt{\overset{n}{\underset{i=1}{\sum }}{\left({\tilde{D}}_{\infty }\left(i\right)-\bar{{\tilde{D}}_{\infty }}\right)}^{2}\overset{n}{\underset{i=1}{\sum }}{\left(P_{\infty }\left(i\right)-\bar{P_{\infty }}\right)}^{2}}}$$

(8) $$D_{COS}\left(d,g\right)=Cos\left({\tilde{D}}_{\infty },P_{\infty }\right)=\frac{\overset{n}{\underset{i=1}{\sum }}{\tilde{D}}_{\infty }\left(i\right)\cdot P_{\infty }\left(i\right)}{\sqrt{\overset{n}{\underset{i=1}{\sum }}{{\tilde{D}}_{\infty }}^{2}\left(i\right)\overset{n}{\underset{i=1}{\sum }}{P_{\infty }}^{2}\left(i\right)}}$$

If a gene has very similar stationary distribution profile with a disease, it may have strong evidence to be the causing gene of the disease. Following this observation, given a disease *d*, its candidate genes were ranked according to the LCC and COS values between their stationary distributions. We referred the two proposed candidate gene prioritization algorithms as DP_LCC and DP_COS respectively, where 'DP' is the abbreviation of '*diffusion profile*'.

### Cross-validation and evaluation criteria

We used two leave-one-out cross-validation methods to validate our algorithm. First is the artificial linkage interval approach, which assumes the singled out interaction is unknown and prioritizes the gene against a set of control genes in the genome. Here the control set consists of the nearest 99 genes around real disease causing genes according to the UCSC refGene table. Actually, there may be few undiscovered disease causing genes in the control set. Second, we used validation against random genes, i.e., in each run, a known disease-gene association is singled out as the test sample against a set of 99 control genes that are selected at random from all genes in the interactome. So, a total of 100 genes (including the real disease-causing gene) are served as test data, and performance of our method is validated by capability to recover the real causing gene from the rest 99 control genes.

We used two measures to evaluate the performance of the proposed method. For each cross-validation run, we calculated the proportion of disease genes that obtain the top prioritization score against the corresponding 99 control genes, and called this measure precision (PRE). Also, given a threshold of rank ratio, we calculated the sensitivity (also called the true positive rate) as the fraction of disease genes ranked above this threshold and the specificity (also called the true negative rate) as the fraction of control genes ranked below the threshold. Varying the threshold of rank ratio from 0 to 1 with the scale 0.01, we are able to draw a receiver operating characteristic (ROC) curve and further calculate the area under this curve (AUC). Clearly, a larger PRE/AUC values indicate a better prediction performance of a prioritization method.

## Results and Discussion

### Effects of parameters

There are two free parameters in our model, the restart probability  γ in the random walk algorithm and the weight parameter  λ in computing the initial probability of a disease. We tested our algorithm on different values of  γ (from 0 to 1 with the step 0.05) and  λ (from 0 to 1 with the step 0.1) and found that the best performance are got at the weight parameterλ=0.5, while the restart parameter  γ only has slight effect on the results (Table 1). In detail, for both DP_LCC and DP_COS, the highest PREs are got at γ=0.25 and λ=0.5, and the largest AUC for DP_LCC is got at γ=0.7 and λ=0.5, while for DP_COS the values are γ=0.5 and λ=0.5, respectively. But in general, PREs and AUCs did not change significantly at different values of  γ, which is in accordance with the observation of Kohler, et al. (2008).

**Table 1:** Prediction accuracies of DP_LCC and DP_COS at  γ = 0.25

γ	DP_LCC		DP_COS	
	**PRE**	**AUC**	**PRE**	**AUC**

0.1	406	0.7591	406	0.7590
0.2	412	0.7616	414	0.7606
0.3	417	0.7625	421	0.7624
0.4	422	0.7638	422	0.7625
0.5	422	0.7634	423	0.7618
0.6	421	0.7627	421	0.7612
0.7	419	0.7619	417	0.7609
0.8	419	0.7620	418	0.7605

**Figure 2:**
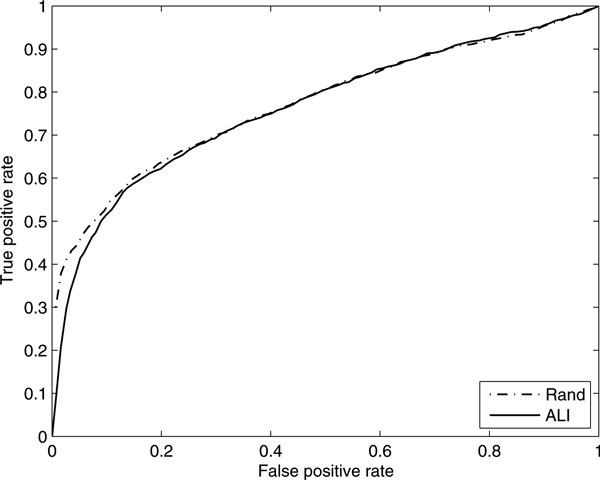
**ROC curves of DP_LCC by different leave-one-out cross-validation methods**. ALI: artificial linkage interval approach; Rand: random genes approach.

We tested our algorithm (DP_LCC) by two leave-one-out cross-validation methods, i.e., artificial linkage interval approach (ALI) and random genes approach (Rand). Results are shown in Figure 2 and suggest a similar performance of DP_LCC by ALI and Rand validations. The only slight difference lies at low values of false positive rates between 0 and 0.1, with a slight superiority of Rand. But when false positive rate increases from 0.1 to 1, two ROC curves are nearly coincident, with AUCs 0.7710 (Rand) and 0.7634 (ALI) respectively. The similar performance of our algorithm by two cross-validation approaches demonstrates that: (i) the control genes by random chosen and from artificial linkage interval are unbiased; (ii) our algorithm is robust to the selection of control set.

The parameter  λ controls the contribution of other related phenotypes to the initial distribution of a phenotype. Large  λ introduce more global dependence of ranking between different correlated phenotypes. When λ=0, the proposed method could be very similar to the RWR algorithm. To investigate the effect of this parameter, we set various values of  λ ranging from 0.1 to 0.9, the performance of our algorithm measured by two evaluation criteria are shown in Table 1. The performances of our algorithm evaluated by two criteria, i.e., LCC and COS, have no significant difference at different values of  λ. The performance is improved when  λ ranges from 0.1 to 0.5, and decreased when  λ is larger than 0.6, especially at 1.0. Therefore, we suggest the  λ value of 0.4 or 0.5.

### Comparison with other diffusion-based methods

To illustrate the utility of the present method, we compared the performances of DP_LCC and DP_COS with two diffusion-based methods, i.e, the RWR and PRINCE on the same gene-phenotype network. Both methods used random walk with restart algorithm to prioritize disease-candidate genes, and achieved relatively better performance compared to linkage-based methods and graph partitioning-based methods [27]. The only difference between RWR and PRINCE is the construction of initial distribution of a disease, where the initial probability vector of RWR was constructed such that equal probability was assigned to each causing gene of a disease, and in PRINCE, the prior information vector was initialized by incorporating disease similarity information by using a logistic function. In our implement, two free parameters *c *and *d *in logistic function are set to -15 and log(9999) respectively, which are in accordance with PRINCE.

We use leave-one-out cross-validation to evaluate the performance of different diffusion-based methods in recovering the gene-phenotype relationship. In each round, a gene-phenotype link was removed, and the rest causal genes and associating phenotypes were used as training set to recover this link. We evaluated the performance of an algorithm in terms of precision (PRE) and the area under ROC curve (AUC) at different values of the rank ratio. The results are shown in Table 2 and Figure 2. As is shown, the proposed method is superior to RWR and PRINCE at nearly all parameter settings in terms of both PRE and AUC. For all these three algorithms, the highest precisions, i.e, 384, 459 and 423 for RWR, PRINCE and our method, respectively, are obtained at γ=0.25. At parameter set λ=0.5, our methods (DP_LCC and DP_COS) successfully ranked 422 and 423 known disease genes as top 1 out of the total 1238 disease-gene interactions. In contrast, only 384 and 359 disease genes were ranked at the top by RWR and PRINCE. While for AUC, the tendency was a little different. AUCs of the RWR algorithm drops with the increase of  γ, and the highest value 0.7549 was achieved at γ=0.1. Similar phenomenon was observed for PRINCE. It is worth mentioning that this phenomenon is slightly different from the result of Kohler [23], who found that the best performance was achieved at γ=0.75. However, the PRINCE algorithm, which also took into consideration the phenotype similarity data, did not achieve high predictive accuracies especially when  γ is larger. This phenomenon may be attributed to the setting of parameters *c *and *d*, which are tuned using cross validation over a totally different dataset, are not quite suitable for the current dataset. We believed that after a careful optimization of the parameters, the PRINCE would achieve a better accuracy than RWR due to the incorporation of phenotype similarities.

**Table 2:** Performances of different algorithms at different values of  γ

Method		0.1	0.2	0.25	0.3	0.4	0.5	0.6	0.7	0.8	0.9
**RWR**	PRE	370	379	**384**	380	380	377	371	370	370	369
	AUC	**0.7549**	0.7549	0.7542	0.7527	0.7507	0.7493	0.7477	0.7465	0.7451	0.7435
**PRINCE**	PRE	350	352	**359**	351	341	329	309	287	263	229
	AUC	**0.7457**	0.7372	0.7309	0.7235	0.7027	0.6758	0.6427	0.6017	0.5429	0.4371
**The present work^a^**	PRE_LCC	405	417	**422**	419	419	417	415	414	413	407
	AUC_LCC	0.7487	0.7616	0.7634	0.7643	0.7659	0.7674	0.7688	**0.7698**	0.7683	0.7694
	PRE_COS	407	421	**423**	419	421	418	412	411	412	406
	AUC_COS	0.7473	0.7604	0.7618	0.7631	0.7637	**0.7644**	0.7638	0.7636	0.7619	0.7606

**^a ^**The pensions are divided by/1238; run time of our algorithm is 38 minutes. Computations were performed on a single processor of a dual-core Intel (R) Core P8700 2.53 GHz and 2 GB GB of shared memory.

**Figure 3:**
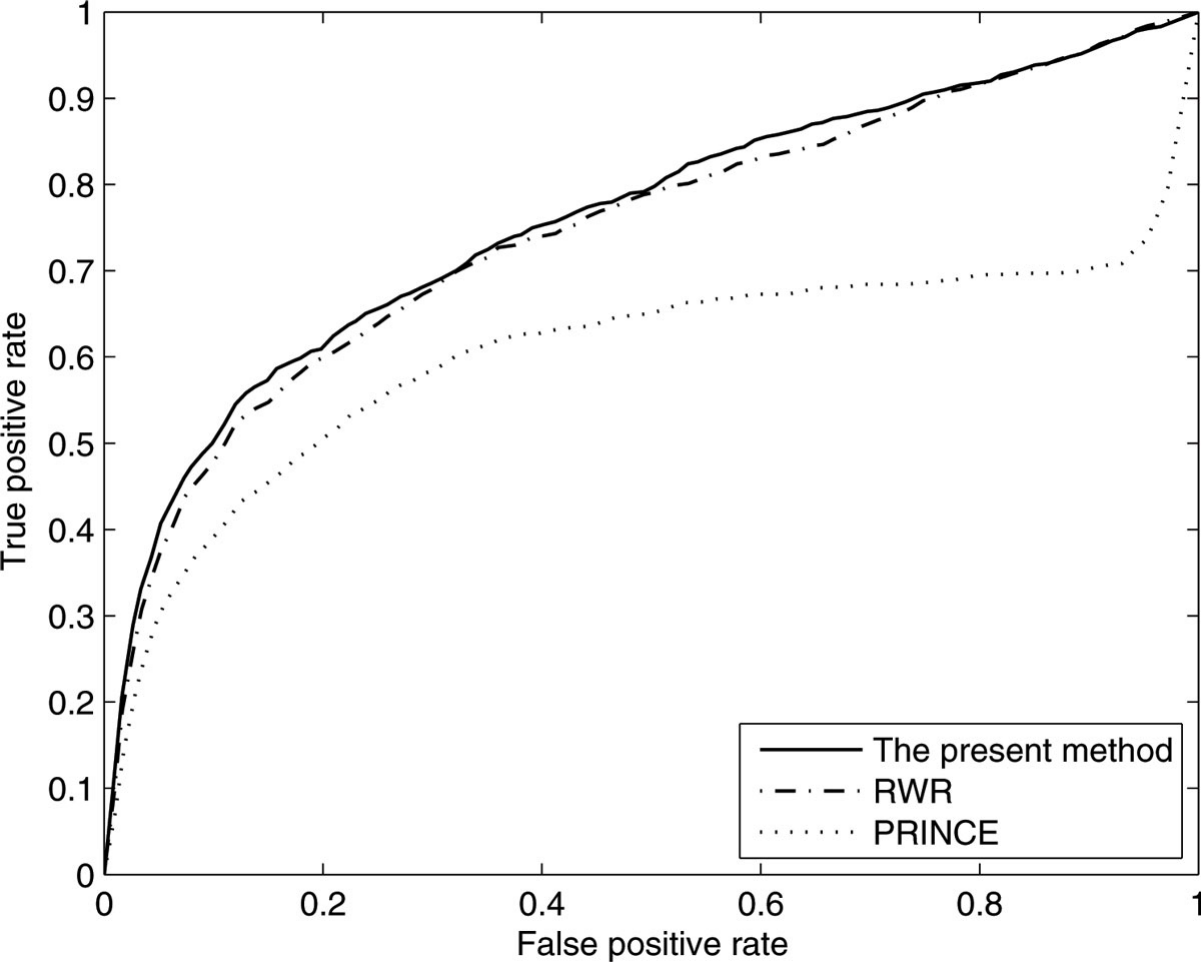
**Comparison of the proposed method with two diffusion-based methods**.

The ROC curves for these three methods at γ=0.7 are shown in Figure 3. We used the receiver operating characteristic (ROC) curve to compare our method with two diffusion-based methods, which plots the sensitivity versus 1-specificity subject to the threshold separating the prediction classes [9]. Sensitivity refers to the percentage of disease genes that were ranked above a particular threshold. Specificity refers to the percentage of non-disease genes ranked below this threshold. As shown in Figure 3, the curve of our algorithm is above those of RWR and PRINCE, which suggest that our algorithm obtained both higher sensitivity and higher specificity. The AUC value of our algorithm is 0.7698, which is much higher than RWR (0.7465) and PRINCE (0.6017).

**Figure 4:**
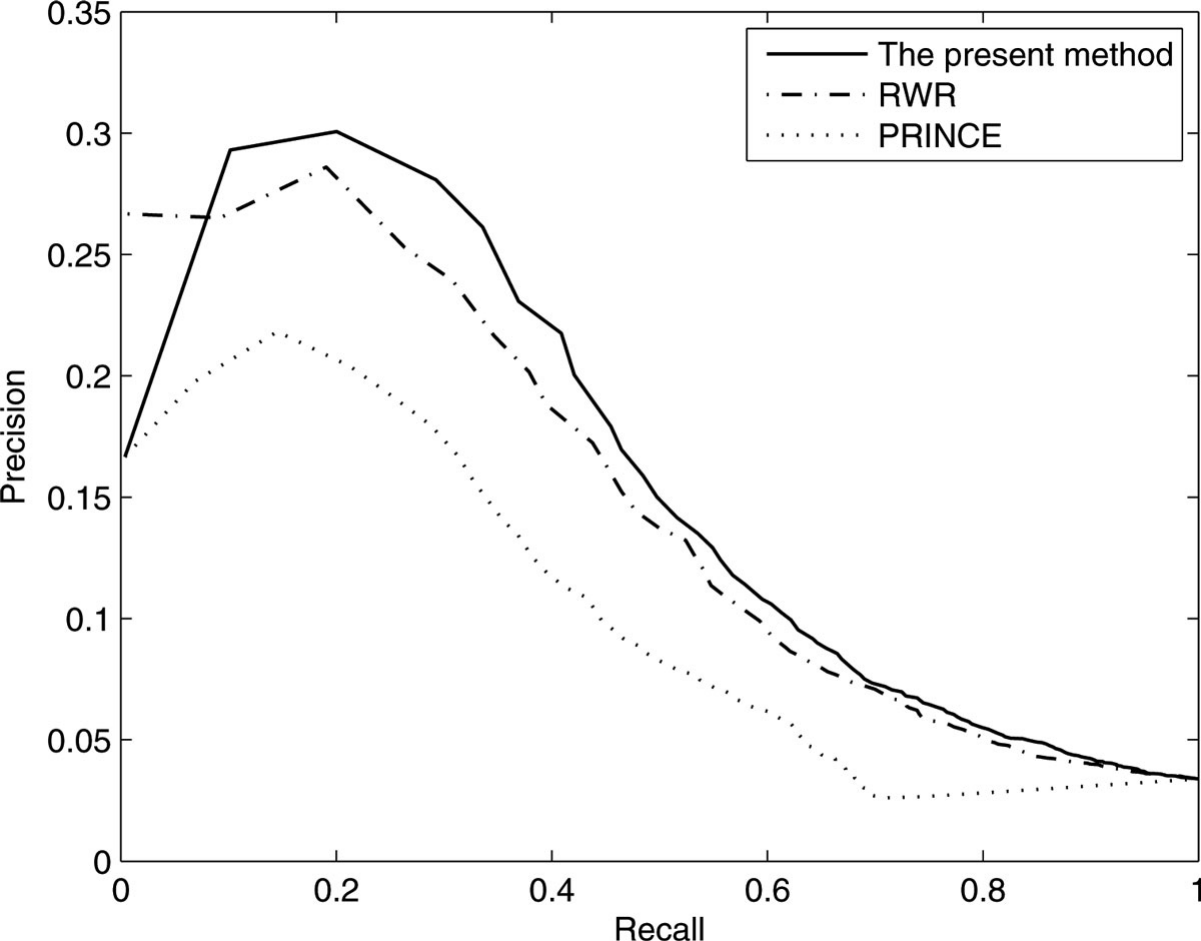
**Precision-recall curves of three diffusion-based methods**.

Another popular method for evaluating the performance of a prioritization method is to consider the precision-recall curve [24,33,34]. Given the association scores calculated for candidate genes, we define positive calls as all genes whose association scores are higher than a certain threshold and define the precision as the proportion of disease genes among the positive calls. And the recall, also called the true positive rate, is defined as the proportion of positively called disease genes among all disease genes. By varying the threshold value, we can compute a series of precision and recall values and obtain a precision-recall (PR) curve. The PR-curves for RWR, PRINCE and our method are shown as Figure 4. As is shown, the curve of our method also lies above those of RWR and PRINCE, which suggests that the performance of our method is superior to the other diffusion-based methods. The superior performance of our model may be attributed to the incorporation of OMIM Phenotype similarities, as well as the global similarity measures between diffusion profiles of diseases and candidate causing genes.

### Predict novel causing genes of Prostate cancer and Alzheimer's disease

After validating our method, we proceeded to execute our algorithm to predict new causing genes of 16 multifactorial diseases that are linked to multiple genomic regions (Table 3). According to the MIM record, all these 16 diseases are associated with more than 10 known valid causing genes locating at different genomic regions. We used our algorithm to predict new causing genes for these 16 diseases, where known causing genes are served as training data and the rest genes in the PPI network are served as candidate disease genes. The top-10 predictions for each disease are shown in Table 3. We selected Alzheimer's disease (MIM: 104300), Prostate cancer (MIM: 176807), Diabetes Mellitus, type 2 (MIM: 125853) as three case studies.

**Table 3:** Top 10 predicted causal genes of 16 multifactoral diseases predicted by DP-LCC

Phenotype Name	PhenoID	Top-10 predictions for each phenotype by our algorithm									
**Alzheimer disease**	**104300**	PAH	TYROBP	TREM2	MAPT	PSEN1	SIGLEC14	CD300E	NCR2	CLEC5A	HLA-DQB1
Breast cancer	114480	PAH	DHCR24	SMYD2	SHISA5	FBXO11	MTM1	TSC1	TSC2	MTMR12	STK11
Colon cancer	114500	MLH1	BRCA2	MSH2	PMS2	VHL	DHCR24	SHISA5	SMYD2	FBXO11	EXO1
**Diabetes mellitus**	**125853**	ABCG8	ABCG5	PPP1R3A	RP1	CFTR	B2M	F2	APC	PLN	CD1E
Gastric cancer	137215	MLH1	PTCH2	MSH2	PMS2	ESR1	SMO	PTCH1	EXO1	PNLIP	IHH
Atrial fibrillation	147050	RAG2	RAG1	CPN1	IGF1R	KL	IL2	IFNA1	CPN2	MEN1	PRSS1
**Prostate cancer**	**176807**	TP53	RET	DHCR24	SMYD2	SHISA5	FBXO11	STK11	NTRK1	CDKN2A	BCL7A
Schizophrenia	181500	PAH	GLO1	TYROBP	TREM2	MAPT	MTM1	MTMR12	SIGLEC14	CD300E	NCR2
Leukemia/lymphoma	190685	POMT1	POMT2	PAH	TYROBP	TREM2	CHRNA1	NSD1	CHRNE	FGFR2	CHRND
Lung cancer	211980	TP53	CDKN2A	SFTPA2	SFTPA1	ZFP91	ZNF227	TBRG1	KIAA1984	CDKN2AIP	ANKRD12
Zellweger syndrome	214100	PEX19	ETFB	ETFA	PEX6	PEX1	PEX12	POMT2	POMT1	SLC25A17	PEX11A
Leukemia	253310	BSCL2	CHRNA1	CHRNG	DOK7	C5orf62	TMEM19	USE1	CHRND	RAPSN	MUSK
Asthma	600807	MARCO	SCGB3A1	RFXAP	RFX5	HPS1	HPS4	RFXANK	IL6	MPO	IL2
Leukemia	601626	MPL	THPO	AMPD1	PDGFRB	HES5	FANCE	FANCD2	BRCA2	ATXN2L	MYH2
Obesity	601665	MC3R	ATRNL1	MC1R	MC5R	ASIP	MC2R	NPY	NPY5R	SIGLEC6	GHRHR
Tuberculosis	607948	NDUFV2	UMOD	HSPA4	AGRP	ASIP	IFNGR2	HPN	TUBA4A	GGCX	LTB

Alzheimer's disease (AD) is the most common cause of dementia in the elderly. It is characterized clinically by progressive memory loss that leads eventually to dementia. As is shown in Table 3, the third prediction for AD is TREM2 (Triggering Receptor Expressed on Myeloid cells), which is a member of the innate immune receptor TREM family [35,36]. It is expressed on the cell surface of the monocyte-macrophage lineage including monocyte derived dendritic cells, osteoclasts and microglia in the Central Nervous System (CNS) [37,38]. Recent researches showed that TREM2 deficiency originates a genetic syndrome characterized by bone cysts and presenile dementia [39]. Another prediction, MAPT, was also a suspicious driver gene for AD [40-43]. Genetic variability at the MAPT locus was shown to be associated with increased risk for the sporadic tauopathies, PSP [44] and corticobasal degeneration [45]. The fifth prediction is PSEN1, which is also a driver gene of AD in the literature. It was reported that mutations in the human presenilin genes (PSEN1 and PSEN2) are associated with early onset familial Alzheimer disease [46].

Diabetes is a chronic condition associated with abnormally high levels of sugar (glucose) in the blood. The disease can be classified into three different categories: the type I, type II and the gestational diabetes. The top 3 predictions of our algorithm for Diabetes mellitus are ABCG8, ABCG5 and PPP1R3A, respectively. The first two genes, i.e., ABCG5 and ABCE8, are ATP-binding cassette transporters that are located in a head-to-head orientation on chromosome 2. The proteins are expressed in the liver, intestine [47,48], and gallbladder epithelial cells [47]. Polymorphisms in ABCG5/ABCG8 genes might contribute to the genetic variation in plasma lipid levels and in cholesterol saturation of the bile [49]. Down-regulation of hepatic and intestinal Abcg5 and Abcg8 expression associated with altered sterol fluxes in rats with streptozotocin-induced diabetes [50]. In addition, defects in Rp1 and PPP1R3A are also causes of susceptibility to diabetes mellitus of type I and II, respectively [51].

Prostate cancer is the most common malignancy in men and the second leading cause of male cancer-related deaths in the Western world. According to the OMIM record, prostate cancer has 25 validate causing genes. Based on these 25 known genes and causing genes of textual related phenotypes, we predicted novel causing genes of prostate cancer using our method (DP_LCC). As is shown in Table 3, the top 3 predictions for prostate cancer are TP53, RET and DHCR24, where TP53 is an important suppressor involved in several types of cancer [52,53]. According to the IARC TP53 Mutation Database [54], inactivating TP53 mutations are detected at frequencies in the range of 10-20% in primary prostate cancer [55]. TP53 was also predicted as the tops by PRINCE. Our second prediction for prostate cancer is RET, which was also found to be overexpressed in high-grade (histopathologically advanced) prostatic intraepithelial neoplasia (PIN) and prostate cancer [56]. So RET was supposed to play a role in the growth of both benign and neoplastic prostate epithelial cells. Another two predicted causing genes, DHCR24 and STK11, were also consistent with the literature. Specifically, DHCR24 is one of androgen receptor-regulated genes implicated in prostate carcinogenesis [57], and STK11 was reported to be inactivated in prostate cancer, through mutation analysis of 24 known cancer genes in the NCI-60 cell line set [58].

## Conclusion

In this paper, a diffusion-based method incorporating pairwise similarities of phenotypes was proposed to prioritize candidate disease genes. The novelty of our method lies in the incorporation of disease phenotypes (OMIM phenotype data) from the literature to the initial state of the RWR, and the usage of global similarity between diffusion profiles of disease and genes. Diffusion profiles of diseases and genes are obtained by walking over the protein-protein interaction network under a given initial distribution, where the initial distribution of a disease was weighted by OMIM Phenotype similarities exceeding a threshold. Then the linear correlation coefficient and cosine of the angle between profiles of a disease and given genes were computed to rank the priorities with the disease. Leave-one-out cross-validation on a benchmark dataset showed that our method achieved a higher precision (measured by PRE and AUC) than existing diffusion-based methods. This result suggests that the proposed algorithm effectively captures the interplay between gene network and phenotype network. We finally predicted causing genes of 16 multifactorial diseases including Prostate cancer and Alzheimer's disease using our algorithm and found that parts of our predictions are in good accordance with current experimental reports.

The superior performance of our method was attributed to the following aspects. First, integration of multiple information sources, especially the phenotype similarity profile data. Second, global similarity measures (linear correlation coefficient and cosine function of the angle) between diffusion profiles of diseases and genes are introduced to prioritize candidate disease genes. In contrast to previous methods that prioritize candidate disease genes through comparing their corresponding components in the diffusion profile of a disease, our global-based method could take into consideration the distribution values of other genes in the PPI network,

Consequently, in the future work, we can integrate some other genomic information to further improve our method, such as gene expression data, functional annotations, pathway membership and so on. Moreover, many researchers pointed out that some diseases might be attributed to a certain protein complexes composed by multiple proteins or a certain pathways. So furthermore attention should be paid on elucidating associations between diseases and protein complexes or pathways.

## Competing interests

The authors declare that they have no competing interests.

## Authors' contributions

JZ conceived of the algorithm and drafted the manuscript. YFQ participated in the algorithm design and the analysis of the data. TGL carried out the programming. XQZ and JW were responsible for the overall design and coordination and helped to revise the manuscript. All authors read and approved the final manuscript.
